# Natural language processing for automated triage and prioritization of individual case safety reports for case-by-case assessment

**DOI:** 10.3389/fdsfr.2023.1120135

**Published:** 2023-02-07

**Authors:** Thomas Lieber, Helen R. Gosselt, Pelle C. Kools, Okko C. Kruijssen, Stijn N. C. Van Lierop, Linda Härmark, Florence P. A. M. Van Hunsel

**Affiliations:** ^1^ Netherlands Pharmacovigilance Centre Lareb, 's-Hertogenbosch, Netherlands; ^2^ Faculty of Social Sciences, Radboud Universiteit, Nijmegen, Netherlands

**Keywords:** prediction model, machine learning, natural language processing, drug safety reports, pharmacovigilance

## Abstract

**Objective:** To improve a previously developed prediction model that could assist in the triage of individual case safety reports using the addition of features designed from free text fields using natural language processing.

**Methods:** Structured features and natural language processing (NLP) features were used to train a bagging classifier model. NLP features were extracted from free text fields. A bag-of-words model was applied. Stop words were deleted and words that were significantly differently distributed among the case and non-case reports were used for the training data. Besides NLP features from free-text fields, the data also consisted of a list of signal words deemed important by expert report assessors. Lastly, variables with multiple categories were transformed to numerical variables using the weight of evidence method.

**Results:** the model, a bagging classifier of decision trees had an AUC of 0.921 (95% CI = 0.918–0.925). Generic drug name, info text length, ATC code, BMI and patient age. were most important features in classification.

**Conclusion:** this predictive model using Natural Language Processing could be used to assist assessors in prioritizing which future ICSRs to assess first, based on the probability that it is a case which requires clinical review.

## 1 Introduction

Before a medicine receives marketing authorization, evidence of its safety and efficacy is limited to the results from clinical trials, where patients are selected carefully and followed up under controlled conditions for a limited period of time. This means that at the time of a medicine’s authorisation, the information about its safety profile is limited. After authorisation the medicine may be used by a large number of patients, for a long period of time, in patients with comorbidities that warrants the use of other medicines. New information on adverse drug reactions (ADRs) may emerge in such circumstances (Stricker and Psaty, 2004; European Medicines Agency, 2022). It is therefore essential that the safety of all medicines is monitored throughout their lifecycle. Pharmacovigilance in this perspective is the *science and activities relating to the detection, assessment, understanding and prevention of adverse effects or any other medicine-related problem* (European Medicines Agency, 2022).

A safety signal in this context is *information on a new or known adverse event that may be caused by a medicine and requires further investigation* (European Medicines Agency, 2021). Safety signals can be detected from a wide range of sources, such as spontaneous reports, clinical studies and scientific literature. Spontaneous reporting systems remain the most efficient and fastest way to get insight in the safety profile of drugs and vaccines (Raine et al., 2007; Lester et al., 2013; Klungel and Pottegård, 2021; Lo Re et al., 2021; Rudolph et al., 2022). Recently, for the COVID-19 vaccines important signals such as thrombosis with thrombocytopenia syndrome for the viral vector vaccines were signalled based on cases (Lane and Shakir, 2022).

Signal detection solely based on the review of individual case safety reports (ICSRs), also called case-by-case analysis or qualitative signal detection, has proven its value (Egberts, 2007). However, it is becoming increasingly time consuming given the growing volumes of data in pharmacovigilance and can become more complex as co-variates may play a role (Egberts, 2007). In the past decades a lot of experience has been gained with the use of different types of disproportionality analyses in signal detection (Bate et al., 1998; van Puijenbroek et al., 2002; Orre et al., 2005; Seabroke et al., 2016). These methods have earned their place in the signal detection process, although they have their limitations. First, they do not take into account the clinically relevant parameters present in the individual reports and should always be followed by clinical review of cases (Scholl, 2022). Secondly, they are prone to certain types of bias (de Geaaf et al., 2003; Pariente et al., 2007) and lastly, a minimum number of cases is needed before a statistical signal can be detected.

Netherlands Pharmacovigilance Centre Lareb relies on both case-by-case analyses and statistical based methods in order to find potential signals. A statistical screening method based on a prediction model is used to identify potential combinations of drugs and adverse drug reactions (ADRs) which require further review (Scholl et al., 2018). In addition, methods such as time-to-onset analysis (Van Holle et al., 2014; Scholl and van Puijenbroek, 2016; Scholl et al., 2019) and topic modelling (Lösch et al., 2022) have been employed, with varying success in finding new signals. The majority of signals is currently still found by cases-by-case analyses in which trained pharmacovigilance assessors, mostly medical doctors and pharmacists, review ICSRs and discuss them during a weekly Signal Detection Meeting (SDM). In order to lower the burden of having to assess all individual cases, Lareb has relied on methods such as triage to decrease the set of reports which needs clinical review by assessors. This triage is performed manually by trained assessors; however, automation of this process could further reduce the workload.

Previously, others have attempted to tackle identifying important cases for signal detection in an automated manner; Munoz et al. (Muñoz et al., 2020). Developed and validated a model predictive of an ICSR’s pharmacovigilance utility based on the United States Food and Drug Administration (FDA) database. The strongest predictors of ICSR inclusion in this study were reporting of a designated medical event (DME) and positive dechallenge. Their validated model showed modest discriminative ability (Muñoz et al., 2020), (Cherkas et al., 2022). Developed a machine learning-based model that can predict the likelihood of a causal association of an observed drug–reaction combination in an ICSR. The model performed well in predicting the causality assessment of drug–event pairs compared with clinical judgment. It should be noted that a causal relationship for a drug-event combination in an ICSR is not the same as having signal value. ICSRs with signal value need to have a certain degree of causality but not all ICSRs with a causal relationship are potential signals. The principle of using elements of information relating to causality assessment in an automated manner was also tested on the French pharmacovigilance database. However, the authors looked at drug-event pairs and not individual ICSRs (Berbain et al., 2020).

Recently, Lareb has published their first efforts to develop a prediction model to identify ICSRs that require clinical review. This was defined by identifying reports which were taken to the Signal Detection Meeting (SDM) and investigating the features of these ICSRs [26]. Most important features in this prediction model were: “absence of ADR in the Summary of product characteristics,” “ADR reported as serious,” “ADR labelled as an important medical event,” “ADR reported by physician” and “positive rechallenge.” An AUC of 0.75 (0.73–0.77) was obtained, which can be seen as moderate model performance (Gosselt et al., 2022).

The aim of this study is to improve a previously developed prediction model that could assist in the triage of individual case safety reports using the addition of features designed from free text fields using natural language processing.

## 2 Materials and methods

### 2.1 Source of data and ICSRs

ICSR reports received from 20 to 03-2019 to 30-04-2022 were extracted from Lareb’s ICSR database. This time period was chosen since different criteria were used to label reports as case or non-case in earlier reports. Reports on vaccines were excluded from our analysis because also for these reports different criteria were used.

#### 2.1.1 Outcome

The binary outcome is defined as ICSRs marked as ‘case’ or ‘non-case’ by scientific assessors. Cases are ICSRs that need in depth clinical review whereas non-case reports can be coded and directly stored in the database without clinical review.

### 2.2 Features

#### 2.2.1 Structured features

The features were chosen from Lareb’s ICSR database based on how informative they were perceived by expert assessors. Of these features all were included into the training data except for: 1) non-informative features, such as the safety report identification number and 2) features with small variances (≤0.0001) with respect to cases or non-cases, as these were unlikely to be useful to discriminate between cases and non-cases.

#### 2.2.2 Natural language processing features

The “additional information” text field contained Dutch descriptions of the ADR that patients experienced and any potential other relevant information such as treatment, contact with a healthcare professional or thoughts on causality made by the reporter (Rolfes et al., 2015). Because the semantic content of this text field could be informative for the prediction, NLP features were extracted from this text field using sklearn (Pedregosa et al., 2011) and nltk (Bird et al., 2009), which are libraries for predictive data analysis and statistical NLP developed for the python programming language.

First, a bag-of-words model was used with tokenization without stemming to assess NLP features, considering the frequency of the words mentioned. Stop words were excluded and a maximum word frequency of 0.2 was set to exclude structured text that was present in many ICSRs. Stop words refer to words that occur commonly in reports, but hold little semantic value. Secondly, the 2000 most frequent words (excluding stop words) in the additional info text field of case and non-case reports were compared. The words that were present in case - but not in non-case reports were subsequently stored on a list. These words were assessed on their usefulness by two independent Lareb pharmacovigilance experts. Also, words that were not yet on the list, but which at least one of the experts considered important, were added. Subsequently, the list was used as a vocabulary for the bag-of-words model and resulting features were appended to the existing feature set.

Lastly, the feature “info_length” was added. This feature represented the number of characters in the additional text field and was based on the assumption that cases would have more text in the additional text field as compared to non-cases.

#### 2.2.3 Weight of evidence feature engineering

Categorical variables that could be equal to more than two different levels were changed into numerical variables using the weight of evidence (WOE) method. WOE is a feature engineering method and has been proven useful for predictive modelling (Dahal et al., 2008; Cao et al., 2021).

The benefits of using the WOE method is that you have one variable instead of multiple dummy variables for each level. This makes it easier to check the feature importance as you have to check only one variable opposed to multiple dummy variables.

The WOE is calculated for each level of a variable. For example, the variable primary_source has multiple levels: $\left\{physician,pharmacist,..,consumer\;or\;other\;non-health\;professional\right\}.$ The WOE can then be calculated using the following formula:

$$WOE=\ln\left\{\frac{P\left(Case\right)}{P\left(Non\;Case\right)}\right\}$$

where $P\left(Case\right)$ and $P\left(Non\;Case\right)$ refer to the proportion of case and non-case reports respectively. If a level has a positive WOE value then the proportion of case reports is higher than the proportion of non-case reports.

### 2.3 Sample size

The dataset contained 49928 reports of which 18,236 (36.5%) were cases. Various resampling strategies (random upsampling of the minority class, random downsampling of the majority class and Synthetic Minority Over-sampling Technique [SMOTE; generating synthetic samples from the minority class]) were tested using imblearn library (Lematre et al., 2017) to balance the number of samples of both classes in the training set.

### 2.4 Missing data

Relatively many features were compulsory for the ICSR reporter to answer, hence little data was missing. Missing data fields can be explained by reports send on paper/letter, or from other origins such as registers. Also, new features have been introduced to the reporting form over time and others have been removed, resulting in features with missing values. Variables containing missing data where age in years, weight in kg and length in cm. These variables were imputed using k nearest neighbor imputation (k = 2 neighbors) using the KNNImputer function from the sklearn package (Pedregosa et al., 2011).

### 2.5 Statistical analyses

#### 2.5.1 Models

Multiple models were trained and tested, including logistic regression, Extreme Gradient Boosting (XGBoost), support vector machine, random forest, decision tree classifier and bagging classifier of decision trees. The bagging classifier was optimized using the parameters :n estimators,max⁡features,and criterion where $n\;estimators=\left\{50,100,200,500,1000\right\}$ refers to the amount of base estimators; $\max features=\left\{10,50,100,120\right\}$ refers to the number of features to draw from the training data to train each base estimator. Lastly; $criterion=\left\{Gini,entropy,\log loss\right\}$ refers to the criterion to be optimized by the model. Best model parameters were chosen using 5-fold cross-validation and found using the RandomizedSearchCV function from the scikit-learn package (Pedregosa et al., 2011).

##### 2.5.1.1 Model training and test set

Firstly, the data was divided into a training set (70%) and a test set (30%) where a fixed proportion case reports/non-case reports in each set was ensured using the RandomOverSampler from the imblearn package (Lematre et al., 2017). Model performance was assessed on the test set using accuracy, precision, recall, f1-score and area under the curve (AUC). The threshold for which the precision and recall were reported was chosen such that Youden’s index was the highest. Feature importance was determined by looking at what features were selected in every base classifier and how important that feature was for the prediction. This was provided by the bagging classifier from scikit-learn package (Pedregosa et al., 2011). These results were then summed for all base classifiers to get an idea of overall feature importance.

All data analysis was performed using Python 3.10.

## 3 Results

The training set contained 34,589 ICSR reports (70%) of which 12,634 (36.5%) were cases. Random upsampling of the minority class in the train set resulted in the best performance (data not shown) and was therefore applied to train the models.

The list of content-related words from the additional_info text that were frequently present in cases and not in non-cases did not improve classification performance of the model. Most words on the list that were acquired using this method were considered irrelevant by two Lareb pharmacovigilance experts. The experts added another 33 words that they considered important in the classification of cases and non-cases. An overview of the final list of words is given in Supplementary Table S1.

In total 175 features were used for prediction. An overview of all the features in the final feature set and a short description of every feature are given in Table 1.

**TABLE 1:** Features in the final data set.

Feature name	Description
Features extracted from database used for prediction	
adr_count	The number of adverse drug reactions (ADR) reported
ATCode	Classification for drugs. The more similar the code, the more closely-related the drugs
comedication_count	The number of drugs that are used in addition to the suspect drug(s)
Dechallenge	If the ADR stops after stopping medication
drug_action_taken	The action that was taken corresponding to the drug when the side effects occurred
drug_brand_name	The brand name of a drug
drug_generic_name	The generic name of a drug
drug_suspect_interacting_count	The number of drugs that are suspected to have caused the adverse drug reaction(s)
Indication	The reason for taking a drug
IME	Important Medical Event, adverse drug reactions that deserve extra attention
Impact based on a Likert scale 1–5	Severity of the adverse drug reaction
latency_start	If the time between starting medication and the onset of the ADR is known, or can be calculated
latency_stop	If the latency between stopping/reducing medication and the onset of ADR is known, or can be calculated
patient_sex	Sex of the patient
patient_age_year	Age of the patient
patient_body_weight_kg	Weight of the patient
patient_height_cm	Height of the patient
primary_source	The professional or consumer that reported a signal
primary_source_function	Specific role of the professional that reported the signal
reaction_outcome	The outcome of the side effect
rechallenge	If after a dechallenge medication is taken again and the same ADR occurs again
Spc	If the ADR is in the Summary of Product Characteristics of the drug
seriousness_case_report_level	Whether the adverse drug reaction was considered to be serious or non-serious. If multiple ADRs were reported, the seriousness of the most serious ADR was reported here
treatment	If the ADR has been treated
medication_previously_used	Whether the drug was used before by the patient according to the additional info text field
primary_source_reaction (1 feature per unique ADR, 99 in total)	The primary ADR of the patient as reported in the additional info text field. Because this is written in natural language text, every type of ADR is a separate feature: if that ADR occurs in the additional info text field or not (hence 1 feature per ADR)
seriousness_death	Whether seriousness_death was set to true in the additional info text field
seriousness_disabling	Whether seriousness_disabling was set to true in the additional info text field
seriousness_hospitalization	Whether seriousness_hospitalization was set to true in the additional info text field
Newly created features	
expert-identified signal words (1 feature per word, 33 in total)	For every expert-identified signal word, if it occurs in the additional info field (hence 1 feature per signal word)
info_length	The total number of characters in the additional info text field

**FIGURE 1:**
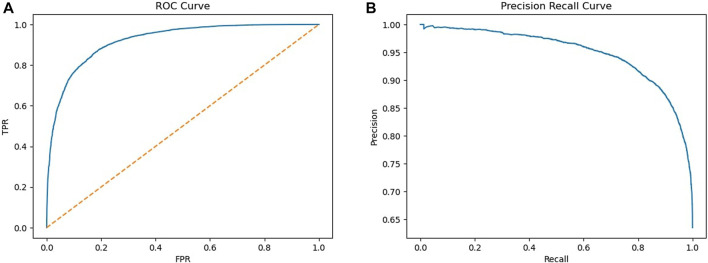
ROC curve **(A)** and precision recall curve **(B)**.

The best-performing model was a bagging classifier of decision trees. The optimal number of base estimators was 100, where each estimator selected a maximum of 100 features out of the 175 for prediction. Performance was good with an f1 score of 0.89 and AUC of 0.921 (95% CI = 0.918–0.925) (Table 2). The ROC curve as well as precision-recall curve showing all cut-off values are presented in Figure 1.

**TABLE 2:** Performance of the bagging classifier on the test set.

Measure	
Accuracy (%)	84.8
Precision (%)	89.3
Recall (%)	86.4
F1 Score	0.89
AUC (95% CI)	0.921 (0.918–0.925)

**FIGURE 2:**
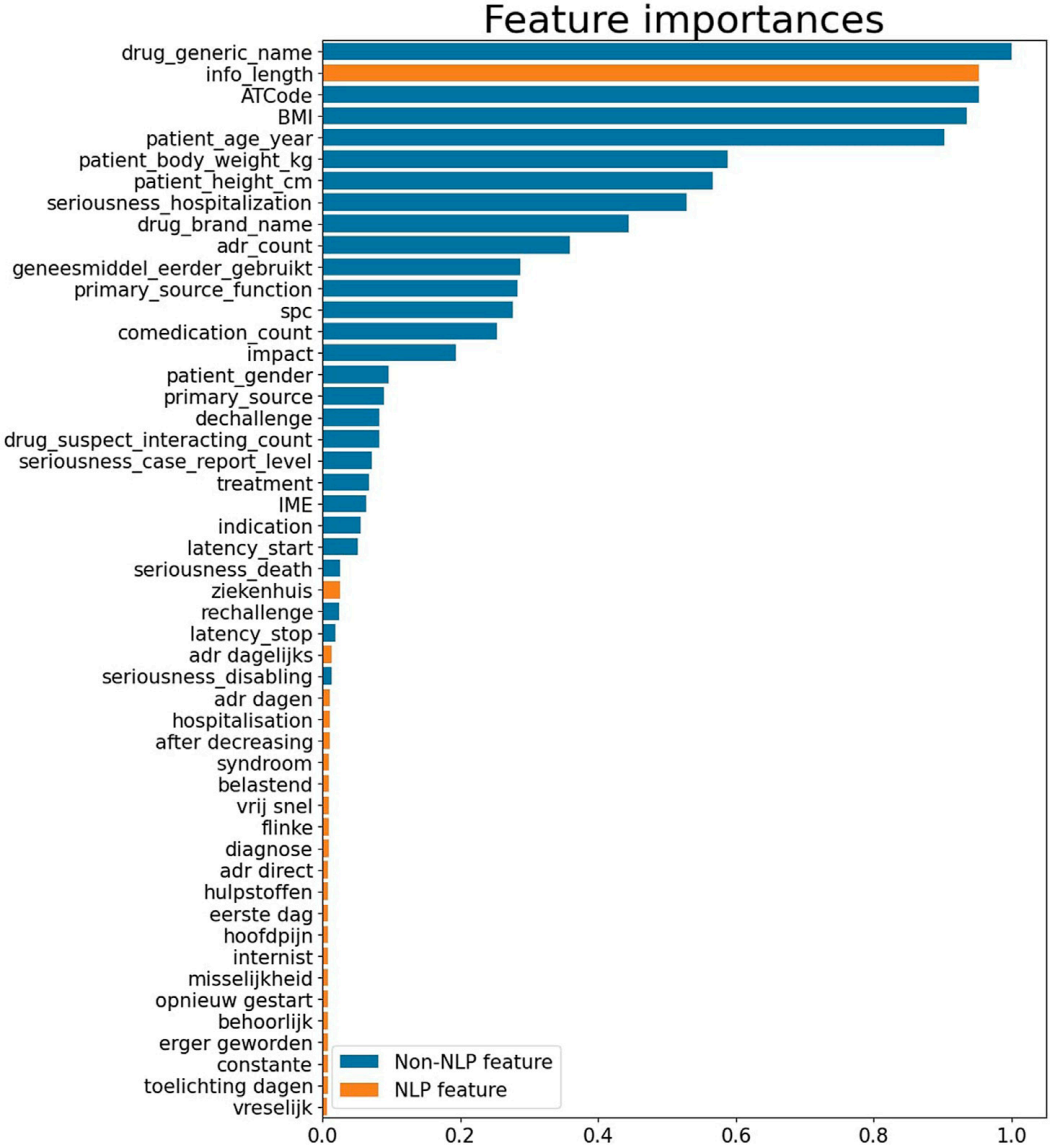
Feature importances of top 50 features. A longer bar indicates greater importance.

The top 50 most important features for prediction are shown in Figure 2.

The plot shows the relative importance of the variables in the model. The direction of the relation is “present.” This means that for example the variable “ziekenhuis” (hospital) can take the values 1 or 0 where 1 means that the presence of the word “ziekenhuis” in the report is predictive of whether a report is a case or non-case. For “patient_gender” a value of 1 refers to male and a 0 to female. The most important features comprised structured fields (non-NLP), describing patient characteristics (e.g., patient BMI or age), or drug descriptions (e.g., drug name, ATC code). (Figure 2). Other structured features that were important where related to the reporter (reporter function) or the ADR (number of ADRs, seriousness, latency, rechallenge/dechallenge). Additionally, the NLP feature “info_length” was of importance. Cases seem to have longer text lengths in the additional information field as compared to non-cases. Other NLP features, or expert signal words also contributed to classification, but were less important.

## 4 Discussion

Predictive modeling can be used for the identification of previously unrecognized risks of medicines in pharmacovigilance ICSRs. These methods have been applied on association level, for instance in “Vigirank” which was developed by the Uppsala Monitoring Centre as a data-driven predictive model for emerging safety signals (Caster et al., 2017). We have developed a prediction model using a combination of structured data fields and unstructured text fields to distinguish case reports from non-case reports with good performance [AUC of 0.92 (95% CI = 0.92–0.93), Precision of 0.89 and recall of 0.86]. The model could be used to assist clinical assessors in ranking the reports on importance. For future reports, the model can predict the probability that the report is a case report based on the different features in the model. A report with long texts in the additional information fields, about a patient with multiple ADRs that are not mentioned in the SmPC, for instance will have a higher probability of being a case report than a report with no additional information, reporting a single ADR that is labelled in the SmPC of the reported drug. Reports with higher probabilities could then be manually reviewed first. Moreover, the cut-off value for which probability the reports should be manually reviewed could be adjusted to the number of reports received. In case the number of reports is increasing and resources are the same, only reports with very high probabilities of being a case report could be reviewed. This is in line with a recent study where a machine learning-based model was developed with the aim to predict the likelihood of a causal association of an observed drug–reaction combination in an ICSR and thus assisting in pharmacovigilance ICSR case processing (Cherkas et al., 2022).

Current model with NLP performed better than our previously developed model that had an AUC of 0.75 (95% CI: 0.73–0.77). In addition, upon visual inspection of the precision and recall plot it was found that the current model also outperformed the previous model which was only based on structured fields (non-NLP features) (Gosselt et al., 2022). From the feature importance plot it can be seen that the feature ‘info_length’ seemed of high importance to distinguish case reports from non-case reports. This is not surprising, since also the follow-up information is added to the additional information, which is more often requested for interesting cases. The list of words defined by pharmacovigilance experts on the other hand, only minimally contributed to the model, however these still contributed more than other techniques that we have tried including: extracting words and compare the frequency between cases and non-cases. This indicates the relevance of domain knowledge for this model. To better understand why some features are high in the feature importance plot, it would be interesting to further assess which reports are classified as case reports. For instance, ATC code or generic drug name are quite high in the list, which indicate that certain drugs are more often reported as case. These could be new drugs for example. Also, in contrast to our previous model, BMI is high in the list. An explanation for this could be that BMI may be related to other features in the dataset, such as the seriousness of an ADR, since high BMI is a risk factor for many complications (Alomar, 2014; Modesto et al., 2020). However, it is also possible that BMI was selected by chance, hence further assessment is required to better understand this result.

Beside the addition of NLP features, the outcome was slightly different defined as compared to our previous model. In our previous model, all reports discussed at a signal detection meeting were classified as “case.” In current study only reports that were potentially important for signal detection were marked as case. This may have created more homogeneous groups of reports which may also have improved the model in classifying the reports. Besides, current dataset contained more reports (49928 as compared to 30424) and the outcome was more balanced with 36.5% cases as compared to 4.7% in our previous study. This lead to a higher number of training instances, which may have also improved the model performances. To limit the scope and complexity of the model, we excluded the “receive_date” of an ICSR in the feature set and made the model ‘static’ for the current time period. However, given that classification criteria may also change over time, it would be interesting to explore the use of continual learning models in future work (Lee and Lee, 2020).

The development of this method is part of a variety of methods such as auto-coding (Létinier et al., 2021; Martin et al., 2022) and automated case classification (Ball et al., 2018) to improve in the processing and signal detection of large amounts of ICSRs in pharmacovigilance databases.

## 5 Limitations

A limitation of our study is that we assessed single extracted NLP words. These single words were not assessed in relation to other words in the text and were taken out of context. It would be interesting to explore part of speech tagging, as this considers the context of the extracted words (Chiche and Yitagesu, 2022). Also, the use of sentiment analysis, which is a tool to extract opinions, perceptions or sentiments from free text (Birjali et al., 2021; Marcec and Likic, 2022). This could be an interesting tool to further explore, because the sentiment of the reporter could be of importance for signal detection. Lastly, the NLP was applied to one unstructured field. The Lareb database has more unstructured fields so in future modelling exercises these other fields could be explored using NLP as well.

Another limitation is that, even though, the definition ‘case report’ as defined by scientific accessors is more specific as compared to all reports discussed at a signal detection meeting, this is still a subjective measure and heterogeneity between assessors may exist. Scientific assessors decide whether the report is a case or non-case based on their interpretation of the report using their expert knowledge. This process is hard to capture using a machine-learning model, which is why this is the best measure we have so far. That is also why the tool could be used to assist the assessors in ranking the reports on importance, while the reports still need clinical review by the assessors.

In conclusion, Natural Language Processing only minimally contributed to the predictive performances of the model. Yet, current model could be used to assist assessors in prioritizing which future ICSRs to assess first, based on the probability that it is a case which requires clinical review.

## Data Availability

The dataset for this study is not publicly available due to Lareb's data protection policy. Access to the data will be granted on reasonable request by the last author.
