# Supplementary material for: Natural language processing for automated triage and prioritization of individual case safety reports for case-by-case assessment
Source: Front Drug Saf Regul. 2023 Feb 7;3:1120135. doi: 10.3389/fdsfr.2023.1120135 (PMC12443081; doi:10.3389/fdsfr.2023.1120135)
Supplement: Supplementary file 1 [file Table1.docx]

Supplementary Material

Natural Language Processing for Automated Signal Classification for Triage of Individual Case Safety Reports

Thomas Lieber^1^*, Helen R. Gosselt^1^ (ORCID ID: 0000-0002-9262-7411), Pelle C. Kools^²^, Okko C. Kruijssen^²^, Stijn N.C. van Lierop^²^, Linda Härmark^1^ (ORCID ID 0000-0002-9314-9298), Florence P.A.M. van Hunsel^1^ (ORCID ID: 0000-0001-8965-3224)

^1^Netherlands Pharmacovigilance Centre Lareb, ‘s-Hertogenbosch, The Netherlands

²Faculty of Social Sciences, Radboud Universiteit, Nijmegen, The Netherlands

*** Correspondence:**T. Lieber

Goudsbloemvallei 7,

5237 MH ‘s-Hertogenbosch

t.lieber@lareb.nl

# Supplementary Data

**Supplementary Table S1.** Words identified by experts from a list of frequent words for cases and not for non-cases or suggested by experts themselves.

|  | **English translation of Dutch text** | **Dutch word** |
| --- | --- | --- |
| 1 | persistent | Aanhoudende |
| 2 | ADR daily | ADR dagelijks |
| 3 | ADR days | ADR dagen |
| 4 | ADR direct | ADR direct |
| 5 | ADR during | ADR gedurende |
| 6 | ADR very | ADR zeer |
| 7 | after decreasing | Na afname |
| 8 | aggressive | Agressief |
| 9 | fairly | Tamelijk |
| 10 | burdensome | Belastend |
| 11 | within hour | Binnen 1 uur |
| 12 | constant | Constante |
| 13 | days stop | Dagen stoppen |
| 14 | diagnosis | Diagnosis |
| 15 | disabling | Onbekwaam maken |
| 16 | doctor | Dokter |
| 17 | first day | Eerste dag |
| 18 | getting worse | Erger worden |
| 19 | considerable | Aanzienlijk |
| 20 | considerably | Flink |
| 21 | fault | Fout |
| 22 | hospitalization | Ziekenhuisopname |
| 23 | excipients | Hulpstoppen |
| 24 | internist | Internist |
| 25 | complaints again | Terugkerende klachten |
| 26 | life threatening | Levensbedreigend |
| 27 | medical fault | Medische fout |
| 28 | nasty | Erg |
| 29 | off label | Off label |
| 30 | off-label | Off-label |
| 31 | discharge letter | Ontslagbrief |
| 32 | restarted | Herstart |
| 33 | passed away | Overleden |
| 34 | couple of weeks | Paar weken |
| 35 | primary source reaction serious | Primary source reaction serieus |
| 36 | stop medication | Stop medicatie |
| 37 | syndrome | Syndroom |
| 38 | comment days | Opmerking dagen |
| 39 | comment serious | Opmerking serieus |
| 40 | comment change | Opmerking verandering |
| 41 | changed | Veranderd |
| 42 | aggravated | Verergerd. |
| 43 | addicted | Verslaafd |
| 44 | addiction | Verslaving |
| 45 | terrible | Erg |
| 46 | terribly | Vreselijk |
| 47 | fairly quickly | Vrij snel |
| 48 | rather immediately | Gelijk |
| 49 | hospital | Ziekenhuis |
